# Falls and Recurrent Falls among Adults in A Multi-ethnic Asian Population: The Singapore Epidemiology of Eye Diseases Study

**DOI:** 10.1038/s41598-018-25894-8

**Published:** 2018-05-15

**Authors:** Wei Dai, Yih-Chung Tham, Miao-Li Chee, Nicholas Y. Q. Tan, Kah-Hie Wong, Shivani Majithia, Charumathi Sabanayagam, Ecosse Lamoureux, Tien-Yin Wong, Ching-Yu Cheng

**Affiliations:** 1 Singapore Eye Research Institute, Singapore National Eye Centre, Singapore, Singapore; 2 Department of Ophthalmology, National University Hospital, Singapore, Singapore; 3 Duke-NUS Medical School, Singapore, Singapore; 4 Department of Ophthalmology, Yong Loo Lin School of Medicine, National University of Singapore, Singapore, Singapore

## Abstract

We evaluated the rate and risk factors associated with falls and recurrent falls in a multi-ethnic Asian population. 10,009 participants aged ≥40 years (mean[SD] age = 58.9[10.4] years) underwent clinical examinations and completed interviewer-administered questionnaires. Participants who self-reported at least one fall or ≥2 falls in past 12 months were defined as fallers and recurrent fallers, respectively. Age-standardized rates for falls and recurrent falls were 13.8% (95%CI, 13.1–14.6%) and 4.6% (95%CI, 4.2–5.1%), respectively. Multivariable analyses showed older age (OR = 1.20; 95%CI, 1.11–1.30), female gender (OR = 1.79; 95%CI, 1.54–2.07), diabetes (OR = 1.22; 95%CI, 1.07–1.40), cardiovascular disease (CVD, OR = 1.37; 95%CI, 1.14–1.65), ≥3 systemic comorbidities (OR = 1.35; 95%CI, 1.09–1.67), lower European Quality of Life-5 Dimensions (EQ-5D) score (OR = 1.36; 95%CI, 1.29–1.44), alcohol consumption (OR = 1.41, 95%CI, 1.11–1.78) and presenting visual impairment (VI, OR = 1.23; 95%CI, 1.02–1.47) were associated with falls. For recurrent falls, female gender (OR = 2.27; 95%CI, 1.75–2.94), diabetes (OR = 1.28; 95%CI, 1.03–1.61), CVD (OR = 2.00; 95%CI, 1.53–2.62), ≥3 systemic comorbidities (OR = 1.69; 95%CI, 1.19–2.39), lower EQ-5D score (OR = 1.47; 95%CI, 1.35–1.59), living in 1–2 room public flat (OR = 1.57; 95%CI, 1.05–2.33), monthly income <2000 Singapore Dollar (OR = 1.62; 95%CI, 1.13–2.31), alcohol consumption (OR = 1.81, 95%CI, 1.23–2.66) and presenting VI (OR = 1.34; 95%CI, 1.01–1.79) were significant risk factors. These findings will be useful for the formulation of fall prevention programs.

## Introduction

Older people are generally at a higher risk of falls, which usually result in adverse outcomes, including serious injuries, fractures, disability, or even death. Falls are also associated with increased burden of healthcare utilization and cost^1–5^. In community-dwelling population aged 65 years or above, 1 in 3 individuals fall at least once every year, and approximately 20% of the fallers have serious injuries^2^. Fall prevention is a leading public concern in many aging populations.

In elderly population, multiple risk factors have been shown to be associated with falls, including visual impairment (VI)^6–8^, impaired balance or gait^9^, systemic disease^10,11^, medication use^12^, and frailty^1,13^. Falls are also associated with psychological impact^14–16^ (e.g. serious psychological distress, fear of falling and depression) and socioeconomic factors^17,18^ (e.g. lower education level, poor house conditions and poor neighborhoods). This further underpins the importance of identifying high fall risk individuals earlier for appropriate administration of prevention and intervention measures.

In recent decades, the elderly population has been expanding rapidly worldwide, especially in Asia. According to the World Health Organization (WHO), by 2050 an estimated 1.5 billion people will be 65 years or older, tripled from estimates (524 million) in 2010^19^. Notably, Asia makes up 60% of the world’s population, and is aging faster than any other region^20^. Despite the rapid aging trend in Asia, there are limited reports on falls among elderly Asians from population-based study. More importantly, no study has evaluated recurrent falls in a multi-ethnic Asian population. Compared to single fall incidents, recurrent falls generally result in even more serious consequences, such as long-term hospitalization, immobility and even death^21^. Hence, comprehensive evaluation of both falls and recurrent falls is important, especially in Asia. Furthermore, to our knowledge, very few Asian population-based studies have comprehensively evaluated risk factors associated with falls and recurrent falls, encompassing demographic, socioeconomic, lifestyle, visual and systemic factors.

The purpose of this study was to evaluate the rate and risk factors of falls and recurrent falls in a multi-ethnic Asian population comprising of Malays, Indians and Chinese, three major ethnic groups in Asia. Findings from this study will provide a unique and comprehensive insight on the trends of falls and recurrent falls in Asians, which will be useful for identifying potential high-risk individuals, and may aid in future formulation of fall prevention programs.

## Methods

### Study Population

The Singapore Epidemiology of Eye Diseases (SEED) Study is a population-based cross-sectional study for three major ethnic groups in Singapore: Malays, Indians and Chinese. Methodology and details of this study have been reported previously^22,23^. In brief, the SEED study was conducted in the southwestern part of Singapore, using a standardized study protocol across the 3 ethnic groups of participants. The data for our study was derived from 3,280 Malay participants (2004 to 2006, response rate 78.7%)^6^, 3,400 Indian participants (2007–2009, response rate 75.6%)^24^ and 3,353 Chinese participants (2009 to 2011, response rate 72.8%)^25^. The study was approved by the Singapore Eye Research Institute Review Board. Written informed consent was obtained from all participants before enrolment and the conduct of the study adhered to the Declaration of Helsinki.

### Clinical Examinations

All participants underwent standardized systemic and ophthalmic examinations at the Singapore Eye Research Institute. Non-fasting blood samples were collected. Diabetes was defined as random glucose ≥11.1 mmol/L, glycated haemoglobin (HbA1c) ≥6.5%, use of diabetic medication, or self-reported history. Hyperlipidaemia was defined as total cholesterol ≥6.2 mmol/L or use of lipid lowering medication. Hypertension was defined as systolic blood pressure (BP) ≥140 mmHg, diastolic BP ≥90 mmHg, antihypertensive drugs usage, or self-reported history of hypertension. Chronic kidney disease (CKD) was defined based on estimated glomerular filtration rate (eGFR) of <60 ml/min/1.73 m^2^, measured from serum creatinine. Cardiovascular disease (CVD) was defined based on self-reported history of angina, stroke or heart attack. Systemic comorbidities were defined as concurrent presence of either diabetes, hyperlipidemia, hypertension, CKD or CVD. The body mass index (BMI) was measured as weight in kilograms divided by height in meters squared. BMI was further divided into subgroups based on the WHO classification: underweight (BMI <18.5), normal (18.5 ≤BMI <25), overweight (25≤ BMI <30) or obese (BMI ≥30).

Visual acuity was measured in each eye separately, using a logarithm of the minimum angle of resolution (logMAR) chart (Lighthouse International, New York, NY) at 4 meters. Presenting visual acuity (PVA) was ascertained with the participants wearing their habitual optical correction (spectacles or contact lenses), if any. If no number could be read at 4 meters, the participant was moved to 3, 2, or 1 meter, consecutively. If no number could be read at all, PVA was assessed as counting fingers, hand movements, perception of light, or no perception of light^24^. In our study, we only used PVA data as PVA is more relevant to the performance of participants’ daily functioning activities, compared to best-corrected visual acuity^6^.

Based on the WHO definition, normal vision was defined as PVA ≤20/60. Low vision was defined as 20/60< PVA ≤20/400. Blindness was defined as PVA >20/400. VI (PVA >20/60) included eyes with low vision or blindness. VI was further defined and categorized based on better or worse eye. For definitions based on better eye, normal vision refers to normal vision in one eye and normal/low vision/blindness in the other eye; low vision refers to low vision in one eye and low vision/blindness in the other eye; blindness refers to blindness in both eyes; VI refers to low vision or worse in both eyes. On the other hand, for definitions based on worse eye, normal vision refers to normal vision in both eyes; low vision refers to low vision in one eye and normal/low vision in the other eye; blindness refers to blindness in one eye and normal/low vision/blindness in the other eye.

### Questionnaire

A detailed interviewer-administered questionnaire was used to collect information including medication use, history of systemic disease, cognitive status, deafness, housing category, living alone, education, monthly income, health-related quality of life (HRQoL), smoking status, alcohol consumption and history of falls. Cognitive assessment was performed for participants aged 60 and above, using the Abbreviated Mental Test (AMT) which consists of 10 questions of general cognitive function. Cognitive impairment was defined as an AMT score of 6 or less out of 10 for the participants with 0 to 6 years of formal education, and an AMT score of 8 or less out of 10 for those with more than 6 years of formal education^26^. Deafness was defined based on self-reported history of hearing loss. Housing category was classified as 1–2 room public flat, 3–4 room public flat, and ≥5 room public flat or private housing. Education level was classified as no formal education, primary education, and secondary education or above. Monthly income level was classified into two groups: <2000 Singapore Dollar (SGD) and ≥2000SGD. Smoking status was classified into current smoker and non-current smoker (including never smoked and past smoker). Alcohol consumption was defined based on self-reported history of having an alcoholic drink at least once a week. The European Quality of Life-5 Dimensions (EQ-5D) Questionnaire was used to measure generic HRQoL, which consists of 5 dimensions: mobility, self-care, usual activities, pain/discomfort and anxiety/depression^6,27^. The EQ-5D score ranges from negative values (e.g. bed-ridden, chronic severe pain) to 1 (representing perfect health)^28^.

### Main Outcomes: Falls and Recurrent Falls

Information about falls was collected by asking the following question - “During the past 12 months, have you had any falls where you have landed on the ground or floor?” If yes, subject was further asked on number of falls in the past 12 months. Faller was defined as an individual who had at least one fall in the past 12 months. Recurrent faller was defined as an individual who had ≥2 falls in the past 12 months.

### Statistical Analysis

All statistical analyses were performed using Stata 13.0 (StataCorp LP, College Station, TX). P < 0.05 indicated statistical significance. In descriptive analyses, unpaired t-test was performed to compare continuous variables between non-fallers and fallers, as well as non-fallers and recurrent fallers; and chi-square test was used for categorical variables. Rate estimates of falls and recurrent falls were calculated and standardized to the Singapore Population Census 2010. Bootstrapping was performed to compare the standardized rate of falls and recurrent falls across 3 ethnicities. Multiple logistic regression was performed to determine the associations between demographic, systemic, socioeconomic factors, VI with falls and recurrent falls respectively, while adjusting for known and potential confounders such as deafness, living status, smoking status and alcohol consumption. In addition, ordinal logistic regression analysis was performed to evaluate the associations of the above factors with frequency of falls which was classified into 4 groups: non-fallers, single fall, fall twice, fall thrice or above.

### Data Availability

The datasets generated during and/or analysed during the current study are not publicly available due to Institute Review Board related matters, but are available from the corresponding author on reasonable request.

## Results

Of the total 10,033 study participants, 10,009 participants with a mean age of 58.9 years (standard deviation = 10.4 years) provided information about history of falls. Of which 1,475 (14.7%) reported having fallen in the past 12 months, 498 (5.0%) reported having recurrent falls in the past 12 months.

Table 1 shows the comparison of demographic, systemic, socioeconomic and lifestyle characteristics between non-fallers and fallers, as well as non-fallers and recurrent fallers. Compared to non-fallers, both fallers and recurrent fallers were more likely to be older, female gender, Indian ethnicity, cognitively impaired, deaf, have higher BMI, lower EQ-5D score, diabetes, hyperlipidemia, hypertension, CKD, CVD, 3 or more systemic comorbidities, live in 1–2 room public flat, live alone, lower education level and low monthly income, but less likely to be current smoker (all P-value < 0.05).

**Table 1:** Characteristics of non-fallers, fallers and recurrent fallers.

Characteristics	Non-fallers (N = 8,534)	Fallers (N = 1,475)	P value*	Recurrent fallers (N = 498)	P value^
Age, years	58.4 (10.2)	61.5 (10.8)	<0.001	61.8 (10.8)	<0.001
Female gender	4,143 (48.6)	935 (63.4)	<0.001	338 (67.9)	<0.001
Ethnicity					
Malay	2,786 (32.7)	480 (32.5)	0.001	162 (32.5)	<0.001
Indian	2,837 (33.2)	555 (37.6)		204 (41.0)	
Chinese	2,911 (34.1)	440 (29.8)		132 (26.5)	
BMI, kg/m^2^	25.3 (4.7)	26.0 (5.0)	<0.001	26.3 (5.3)	<0.001
Diabetes	2,407 (29.6)	547 (39.1)	<0.001	204 (43.4)	<0.001
Hyperlipidemia	3,707 (44.8)	690 (48.7)	0.007	235 (49.8)	0.035
Hypertension	5,150 (60.5)	1,004 (68.3)	<0.001	347 (70.0)	<0.001
Chronic kidney disease	976 (11.9)	235 (16.8)	<0.001	90 (19.2)	<0.001
Cardiovascular disease	855 (10.0)	225 (15.3)	<0.001	105 (21.2)	<0.001
Systemic comorbidities^#^					
No systemic disease	1,785 (22.3)	215 (15.8)	<0.001	64 (14.2)	<0.001
Any 1 systemic disease	2,425 (30.2)	371 (27.3)		106 (23.6)	
Any 2 systemic diseases	2,076 (25.9)	351 (25.9)		111 (24.7)	
≥3 systemic diseases	1,732 (21.6)	421 (31.0)		169 (37.6)	
Cognitive impairment^†^	493 (13.4)	172 (20.5)	<0.001	63 (22.1)	<0.001
Deafness	29 (0.3)	15 (1.0)	<0.001	10 (2.0)	<0.001
EQ-5D score	0.87 (0.2)	0.77 (0.2)	<0.001	0.72 (0.3)	<0.001
Housing categories					
1–2 room public flat	579 (6.8)	156 (10.6)	<0.001	66 (13.3)	<0.001
3–4 room public flat	5,302 (62.2)	900 (61.1)		309 (62.1)	
≥5 room public flat/private housing	2,643 (31.0)	418 (28.4)		123 (24.7)	
Living alone	399 (4.7)	95 (6.5)	0.004	37 (7.4)	0.005
Education level					
No formal education	1,859 (21.8)	472 (32.1)	<0.001	173 (34.8)	<0.001
Primary education	3,225 (37.9)	497 (33.8)		180 (36.2)	
Secondary education or above	3,435 (40.3)	503 (34.2)		144 (29.0)	
Monthly income <2000SGD	6,388 (76.5)	1,227 (84.4)	<0.001	443 (89.9)	<0.001
Current smoker	1,428 (16.8)	170 (11.5)	<0.001	57 (11.5)	0.002
Alcohol consumption	730 (8.6)	117 (7.9)	0.422	42 (8.4)	0.916

N = number of participants, SD = standard deviation; BMI = the body mass index; EQ-5D = the European Quality of Life-5 Dimensions; SGD = the Singapore Dollar.

Data presented as number (%), except for age, BMI and EQ-5D score which are expressed as mean (standard deviation).

^#^Systemic comorbidities were classified based on the concurrent presence of two or more systemic conditions such as diabetes, hyperlipidemia, hypertension, chronic kidney disease or cardiovascular disease.

^*^P value refers to comparison between non-fallers and fallers.

^^^P value refers to comparison between non-fallers and recurrent fallers.

All P values were obtained with unpaired-t test for continuous variables, chi-square test for categorical variables.

^**†**^Cognitive impairment data was only available for subjects aged 60 years and above.

Table 2 shows the rate of falls and recurrent falls. Overall age-standardized rate for falls and recurrent falls are 13.8% (95% confidence interval (CI), 13.1–14.6%) and 4.6% (95%CI, 4.2–5.1%), respectively. The rate of falls and recurrent falls across 3 ethnicities increases with age (per decade older, all P-trend ≤0.001), with age ≥70 years having the highest rate for falls and recurrent falls, compared to other 3 younger age groups. Indians have the highest age-standardized rate for falls (15.1%; 95%CI, 13.8–16.5%) and recurrent falls (5.6%; 95%CI, 4.8–6.5%), compared to Chinese and Malays (all P < 0.001).

**Table 2:** Rate of falls and recurrent falls.

	Number of individuals	Falls			Recurrent falls		
		Number of cases	Unadjusted rate, %	Age-standardized^^^ rate, % (95% CI)	Number of cases	Unadjusted rate, %	Age-standardized^^^ rate, % (95% CI)
Overall	10,009	1,475	14.7	13.8 (13.1–14.6)	498	5.0	4.6 (4.2–5.1)
Age groups							
40–49 years	2,473	280	11.3		90	3.6	
50–59 years	3,142	377	12.0		129	4.1	
60–69 years	2,555	423	16.6		132	5.2	
≥70 years	1,838	395	21.5		147	8.0	
			P-trend < 0.001			P-trend < 0.001	
By ethnicity:							
Malay	3,266	480	14.7	13.1 (11.8–14.6)**	162	5.0	4.5 (3.7–5.3)**
40–49 years	813	98	12.1		34	4.2	
50–59 years	952	99	10.4		33	3.5	
60–69 years	777	126	16.2		42	5.4	
≥70 years	724	157	21.7		53	7.3	
			P-trend < 0.001			P-trend = 0.001	
Indian	3,392	555	16.4	15.1 (13.8–16.5)	204	6.0	5.6 (4.8–6.5)
40–49 years	957	111	11.6		40	4.2	
50–59 years	1,077	173	16.1		65	6.0	
60–69 years	883	164	18.6		55	6.2	
≥70 years	475	107	22.5		44	9.3	
			P-trend < 0.001			P-trend < 0.001	
Chinese	3,351	440	13.1	12.2 (11.1–13.5)**	132	3.9	3.5 (2.9–4.2)**
40–49 years	703	71	10.1		16	2.3	
50–59 years	1,113	105	9.4		31	2.8	
60–69 years	895	133	14.9		35	3.9	
≥70 years	639	131	20.5		50	7.8	
			P-trend < 0.001			P-trend < 0.001	

95% CI = 95% confidence interval.

^^^Rate estimates were age-standardized to the Singapore Population Census 2010. ^**^Denotes statistically significant difference (P < 0.001) compared to Indian group.

The associations between demographic, systemic, socioeconomic factors and falls or recurrent falls in multivariable models are shown in Table 3. When adjusted for age, gender, ethnicity, presenting VI (better eye), BMI, living alone, EQ-5D score, deafness, socioeconomic factors, systemic disease/comorbidity, current smoker and alcohol consumption, significant factors associated with falls include older age (per decade increase, odds ratio (OR) = 1.20; 95%CI,1.11–1.30), female gender (OR = 1.79; 95%CI,1.54–2.07), diabetes (OR = 1.22; 95%CI,1.07–1.40), CVD (OR = 1.37; 95%CI,1.14–1.65), 3 or more systemic comorbidities (OR = 1.35; 95%CI,1.09–1.67), lower EQ-5D score (OR = 1.36; 95%CI,1.29–1.44) and alcohol consumption (OR = 1.41; 95%CI, 1.11–1.78). On the other hand, significant factors associated with recurrent falls include female gender (OR = 2.27; 95%CI,1.75–2.94), diabetes (OR = 1.28; 95%CI,1.03–1.61), CVD (OR = 2.00; 95%CI,1.53–2.62), 3 or more systemic comorbidities (OR = 1.69; 95%CI, 1.19–2.39), lower EQ-5D score (OR = 1.47; 95%CI,1.35–1.59), living in 1–2 room public flat (compared to ≥5 room public flat/private housing, OR = 1.57; 95%CI,1.05–2.33), monthly income <2000SGD (OR = 1.62; 95%CI,1.13–2.31) and alcohol consumption (OR = 1.81; 95%CI, 1.23–2.66).

**Table 3:** The associations between demographic, systemic, socioeconomic characteristics with falls/recurrent falls.

Characteristics	Falls		Recurrent falls	
	OR (95% CI)^*^	P value	OR (95% CI)^*^	P value
Age (per decade older)	1.20 (1.11, 1.30)	<0.001	1.13 (0.99, 1.28)	0.066
Female gender	1.79 (1.54, 2.07)	<0.001	2.27 (1.75, 2.94)	<0.001
Ethnicity				
Malay	Reference		Reference	
Indian	1.10 (0.94, 1.29)	0.216	1.24 (0.96, 1.60)	0.105
Chinese	1.10 (0.93, 1.31)	0.254	1.25 (0.94, 1.67)	0.131
BMI				
Normal	Reference		Reference	
Underweight	0.87 (0.63, 1.20)	0.387	0.90 (0.53, 1.55)	0.708
Overweight	1.00 (0.87, 1.15)	0.957	0.97 (0.77, 1.23)	0.826
Obese	1.10 (0.92, 1.32)	0.301	1.19 (0.89, 1.59)	0.240
Systemic disease				
Diabetes	1.22 (1.07, 1.40)	0.004	1.28 (1.03, 1.61)	0.028
Hypertension	1.09 (0.95, 1.26)	0.230	1.13 (0.89, 1.45)	0.311
Hyperlipidaemia	0.92 (0.81, 1.05)	0.202	0.91 (0.73, 1.12)	0.355
Chronic kidney disease	1.01 (0.83, 1.21)	0.950	1.10 (0.82, 1.47)	0.539
Cardiovascular disease	1.37 (1.14, 1.65)	0.001	2.00 (1.53, 2.62)	<0.001
Systemic Comorbidities^#^				
No systemic disease	Reference		Reference	
Any 1 systemic disease	1.15 (0.95, 1.39)	0.153	1.08 (0.78, 1.51)	0.638
Any 2 systemic diseases	1.11 (0.91, 1.36)	0.302	1.12 (0.80, 1.59)	0.505
≥3 systemic diseases	1.35 (1.09, 1.67)	0.005	1.69 (1.19, 2.39)	0.004
P-trend	—	0.010	—	0.001
Deafness	1.09 (0.48, 2.52)	0.834	1.93 (0.69, 5.39)	0.206
EQ-5D score (per SD decrease)	1.36 (1.29, 1.44)	<0.001	1.47 (1.35, 1.59)	<0.001
Housing categories				
≥5 room public flat/private housing	Reference		Reference	
1–2 room public flat	1.25 (0.97, 1.62)	0.087	1.57 (1.05, 2.33)	0.028
3–4 room public flat	0.94 (0.81, 1.09)	0.392	1.13 (0.83, 1.55)	0.438
Living alone	1.11 (0.85, 1.44)	0.446	1.11 (0.73, 1.70)	0.616
Education level				
No formal education	Reference		Reference	
Primary education	0.94 (0.79, 1.10)	0.430	1.02 (0.78, 1.33)	0.897
Secondary education or above	1.10 (0.91, 1.33)	0.324	1.13 (0.83, 1.55)	0.438
Monthly income				
≥2000SGD	Reference		Reference	
<2000SGD	1.10 (0.91, 1.33)	0.322	1.62 (1.13, 2.31)	0.009
Current smoker	0.93 (0.75, 1.14)	0.479	1.05 (0.74, 1.50)	0.771
Alcohol consumption	1.41 (1.11, 1.78)	0.004	1.81 (1.23, 2.66)	0.003

OR = odds ratio, 95% CI = 95% confidence interval, SD = standard deviation, BMI = the body mass index, EQ-5D = European Quality of Life-5 Dimensions, SGD = the Singapore Dollar.

^*^Model adjusted for age, gender, ethnicity, presenting visual impairment (based on better eye), BMI, living alone, education, housing categories, income, deafness, EQ-5D score, systemic diseases/comorbidities, current smoker and alcohol consumption.

^#^Systemic comorbidities were classified based on the concurrent presence of two or more systemic conditions, namely, diabetes, hyperlipidemia, hypertension, chronic kidney disease or cardiovascular disease.

The evaluation of systemic comorbidities as exposure of interest was performed in a separate model where diabetes, hypertension, hyperlipidaemia, chronic kidney disease and cardiovascular disease were not included in the model.

The associations between VI with falls and recurrent falls are shown in Table 4. When adjusted for age, gender, ethnicity, BMI, EQ-5D score, deafness, living alone, socioeconomic factors, systemic diseases, current smoker and alcohol consumption, low vision (in better eye) (OR = 1.21; 95%CI, 1.00–1.46) was associated with falls. In addition, VI (in better eye) was also associated with both falls (OR = 1.23; 95%CI,1.02–1.47) and recurrent falls (OR = 1.34; 95%CI,1.01–1.79). Blindness (in worse eye) was associated with both falls (OR = 1.47; 95%CI,1.13–1.91) and recurrent falls (OR = 1.55; 95%CI,1.04–2.32).

**Table 4:** The associations between visual impairment and falls/recurrent falls.

Visual impairment‡	Falls			Recurrent falls		
	OR (95% CI)			OR (95% CI)		
	Number of cases (%)	Model 1^#^	Model 2^^^	Number of cases (%)	Model 1^#^	Model 2^^^
PVA in better eye						
Normal	1,244 (14.0)	Reference	Reference	411 (5.1)	Reference	Reference
Visually impaired	231 (21.1)	1.26 (1.07, 1.49)*	1.23 (1.02, 1.47)*	87 (9.2)	1.40 (1.08, 1.80)*	1.34 (1.01, 1.79)*
PVA in better eye						
Normal	1,244 (14.0)	Reference	Reference	411 (5.1)	Reference	Reference
Low vision	214 (20.6)	1.24 (1.05, 1.47)*	1.21 (1.00, 1.46)*	80 (8.9)	1.37 (1.05, 1.78)*	1.32 (0.98, 1.78)
Blindness	17 (29.8)	1.69 (0.94, 3.02)	1.46 (0.78, 2.75)	7 (14.9)	1.85 (0.81, 4.25)	1.65 (0.66, 4.12)
PVA in worse eye						
Normal	991 (13.6)	Reference	Reference	329 (5.0)	Reference	Reference
Low vision	375 (16.5)	0.99 (0.86, 1.13)	0.97 (0.83, 1.13)	125 (6.2)	0.96 (0.77, 1.20)	0.91 (0.71, 1.18)
Blindness	107 (24.7)	1.54 (1.21, 1.96)**	1.47 (1.13, 1.91)*	43 (11.7)	1.79 (1.25, 2.55)*	1.55 (1.04, 2.32)*

OR = odds ratio, 95% CI = 95% confidence interval, PVA = presenting visual acuity.

^#^Model 1 adjusted for age, gender and ethnicity.

^^^Model 2 adjusted for age, gender, ethnicity, BMI, EQ-5D score, deafness, living alone, education, housing categories, income, diabetes, hypertension, hyperlipidaemia, chronic kidney disease, cardiovascular disease, current smoker and alcohol consumption.

^**‡**^Visual impairment was defined based on the WHO definition: low vision was defined as 20/60 < PVA ≤ 20/400, blindness was defined as PVA > 20/400. Visually impaired encompasses both low vision and blindness.

^*^Denotes P-value < 0.05, **denotes P-value < 0.001.

## Discussion

In this multi-ethnic Asian population, we observed female gender, systemic comorbidities, lower EQ-5D score, alcohol consumption and VI were associated with both falls and recurrent falls. Indians have slightly higher rate of falls and recurrent falls compared to Chinese and Malays. To the best of our knowledge, this is the first population-based study which evaluated the trends and associated risk factors for falls and recurrent falls in a multi-ethnic Asian population. These findings will provide useful information in formulation of fall prevention programs among elderly in Asia.

Although previous studies provided some information on risk factors for falls in some specific fields, e.g. VI^6^, bone and joint disease^9^ and depression^16^, there are still limited reports with comprehensive evaluation of risk factors for falls and recurrent falls in Asian community-dwelling elderly people. For the first time, we also demonstrated having 3 or more systemic comorbidities was associated with higher risk for both falls and recurrent falls in a population-based study. Consistent with our findings, previous studies also demonstrated that VI^29^, female gender^30^, older age^31^, systemic disease^32^ and low HRQoL^6^ significantly increased the risk for falls. However, unlike some previous studies^33,34^, deafness is not a risk factor for falls in our study. Two reasons might explain the lack of positive association between deafness and falls: first, we used self-reported information about deafness which may inadvertently rule out cases with low-degree hearing loss, thus may not be entirely accurate. Second, the number of deafness (N = 44) is very small and thus insufficient statistical power to detect significant association.

Compared with current guidelines and fall assessment tools developed in western countries, our findings in Asian population similarly found that older age, systemic diseases and VI were risk factors for falls. In addition, our study further reported that having 3 or more systemic comorbidities was associated with higher risk for both falls and recurrent falls; socio-economic factors such as housing condition and monthly income status are also important determinants for recurrent falls. These aspects have yet been widely reported in Asians and highlighting the potential usefulness of further incorporating both systemic and socio-economic factors into fall assessment. According to the American Geriatrics Society/British Geriatrics Society Clinical Practice Guideline for Prevention of Falls in Older Persons (2010), muscle strength, gait and balance were also indicated as predictor for falls. Nevertheless, these factors were not measured and thus not evaluated in our study.

In our study, age-standardized rates for falls and recurrent falls were 13.8% and 4.6%, respectively. These rates are comparatively lower than previous population-based studies which reported fall and recurrent fall rates ranging from 17.6–28.4%^10,11,35^ and 9.1–10.4%^36,37^, respectively. This difference may be explained by the younger participants in our sample (aged ≥40 years), compared to previous studies which comprised of older adults (aged ≥60 years). In addition, the lower rates observed in our study may also be due to the self-report collection method employed in our study as compared to other previous studies which used the more accurate method of fall diaries to document history of falls. The difference in methods in documenting history of falls between our study and previous studies which used fall diaries prohibits direct and accurate comparisons. In this study, we also observed Indians to have slightly higher rate of falls and recurrent falls, compared to Malays and Chinese. This may be in part explained by the ethnic differences in risk factor profiles for falls. For example, compared to Malays and Chinese, Indians were more likely to have diabetes, CVD and alcohol consumption, which are significant risk factors for falls and recurrent falls. Meanwhile, we observed females to have higher risk for both falls and recurrent falls, this may be due to females are more likely to suffer from osteoporosis^38^, urinary incontinence^10^ and weaker muscle strength^9^. In the socio-economic aspects, monthly income <2000 SGD and living in 1–2 room public flat were associated with higher risk of recurrent falls. On the other hand, we found that reduced EQ-5D score was associated with falls and recurrent falls. In addition, when further evaluating the individual components of EQ-5D, it was observed that poorer mobility score (per unit change, OR = 1.46, 95%CI, 1.24–1.74; OR = 2.02, 95%CI, 1.57–2.60, respectively), higher pain/discomfort score (per unit change, OR = 1.50, 95%CI, 1.34–1.69; OR = 1.51, 95%CI, 1.25–1.83, respectively) and higher anxiety/depression score (per unit change, OR = 1.29, 95%CI, 1.14–1.48; OR = 1.46, 95%CI, 1.22–1.74, respectively) were the domains associated with both falls and recurrent falls (all P < 0.001, data not shown in tables). These collectively indicate that the physical, mental and overall well-being are all important determinants for falls and recurrent falls as well.

When evaluating frequency of falls as ordinal outcome (Supplementary Table 1), we similarly observed that, older age, female gender, VI (based on better eye), diabetes, CVD, 3 or more systemic comorbidities, lower EQ-5D score and alcohol consumption were associated with higher frequency of falls. In our study, we also explored the interactions between exposure variables, e.g. VI and systemic comorbidity, VI and systemic disease, EQ-5D score and systemic disease. However, no significant interactions were observed in the multivariable model. In multivariable models, some of the risk factors were more prominently observed among older subjects (age ≥60 years), e.g. low socioeconomic status and CVD; however, no significant interaction was observed between age and these factors.

Some fall prevention measures may be inferred from our findings. Firstly, the assessment and interventions for falls should be multifactorial because multiple risk factors are associated with falls, e.g. heavy drinkers, people with 3 or more systemic comorbidities, low HRQoL and low socioeconomic status. Secondly, active screening and appropriate treatment for VI may be recommended for elderly as part of a holistic approach to intervene and prevent fall incidents. In this regard, a prospective cohort study concluded that recent development of VI increased the risk of subsequent falls in the next 5 years^7^. Interventions and treatment on VI have also been shown to reduce the number of falls, especially on preventable and treatable VI causes, such as refractive error and cataract^39,40^. Taken together, this further emphasized the importance of incorporating vision screening/assessment into the collective intervention strategy in preventing and reducing fall incidents.

The strengths of this study include the large sample size from a multi-ethnic Asian population-based study. In addition, we used standardized clinical examinations and questionnaire to collect data about potential risk factors across three ethnic groups, thus allow us to conduct a direct and comprehensive evaluation of risk factors associated with falls and recurrent falls, encompassing demographic, socioeconomic, lifestyle, visual and systemic factors. However, this study also has a few limitations. First, we only collected data on frequency of falls, but did not further collect information on fall-related consequences, such as fractures, hospital admission and disability. Second, as fall information was acquired self-reportedly via questionnaire, there might be recall bias which may result in under or over-reporting of the frequency of falls. Third, although mobility self-assessment was obtained in our study via the EQ-5D questionnaire, measurements on physical function such as muscle strength, gait and balance which are also potential predictors for falls, were not collected in our study.

In conclusion, in this multi-ethnic Asian population, female gender, systemic comorbidities, lower EQ-5D score, alcohol consumption and VI were associated with both falls and recurrent falls. These findings may aid in future formulation of fall prevention programs.

## Electronic supplementary material

Supplementary Information

## References

[1] Kojima G (2015). Frailty as a Predictor of Future Falls Among Community-Dwelling Older People: A Systematic Review and Meta-Analysis. Journal of the American Medical Directors Association.

[2] Tinetti ME, Speechley M, Ginter SF (1988). Risk factors for falls among elderly persons living in the community. The New England journal of medicine.

[3] Reinsch S, MacRae P, Lachenbruch PA, Tobis JS (1992). Attempts to prevent falls and injury: a prospective community study. The Gerontologist.

[4] Tinetti ME (1994). A multifactorial intervention to reduce the risk of falling among elderly people living in the community. The New England journal of medicine.

[5] Fife D, Barancik JI (1985). Northeastern Ohio Trauma Study III: incidence of fractures. Annals of emergency medicine.

[6] Lamoureux EL (2008). Visual impairment, causes of vision loss, and falls: the singapore malay eye study. Investigative ophthalmology & visual science.

[7] Hong T, Mitchell P, Burlutsky G, Samarawickrama C, Wang JJ (2014). Visual impairment and the incidence of falls and fractures among older people: longitudinal findings from the Blue Mountains Eye Study. Investigative ophthalmology & visual science.

[8] Patino CM (2010). Central and peripheral visual impairment and the risk of falls and falls with injury. Ophthalmology.

[9] Muraki S (2013). Risk factors for falls in a longitudinal population-based cohort study of Japanese men and women: the ROAD Study. Bone.

[10] Gale CR, Cooper C, Aihie Sayer A (2016). Prevalence and risk factors for falls in older men and women: The English Longitudinal Study of Ageing. Age and ageing.

[11] Sibley KM, Voth J, Munce SE, Straus SE, Jaglal SB (2014). Chronic disease and falls in community-dwelling Canadians over 65 years old: a population-based study exploring associations with number and pattern of chronic conditions. BMC geriatrics.

[12] Callisaya ML, Sharman JE, Close J, Lord SR, Srikanth VK (2014). Greater daily defined dose of antihypertensive medication increases the risk of falls in older people–a population-based study. Journal of the American Geriatrics Society.

[13] Tom SE (2013). Frailty and fracture, disability, and falls: a multiple country study from the global longitudinal study of osteoporosis in women. Journal of the American Geriatrics Society.

[14] Tran TV, Phan PT (2018). Serious psychological distress, sex, and falls among the elderly. Journal of women & aging.

[15] Mat S, Ng CT, Fadzil F, Rozalli FI, Tan MP (2017). The mediating role of psychological symptoms on falls risk among older adults with osteoarthritis. Clinical interventions in aging.

[16] Kwan MM, Lin SI, Close JC, Lord SR (2012). Depressive symptoms in addition to visual impairment, reduced strength and poor balance predict falls in older Taiwanese people. Age and ageing.

[17] Ahmad Kiadaliri A, Turkiewicz A, Englund M (2018). Educational inequalities in falls mortality among older adults: population-based multiple cause of death data from Sweden. Journal of epidemiology and community health.

[18] Ryu E (2017). Individual housing-based socioeconomic status predicts risk of accidental falls among adults. Annals of epidemiology.

[19] Salive ME (1992). Functional blindness and visual impairment in older adults from three communities. Ophthalmology.

[20] Grover S (2015). Aging population in Asia: Are we preparing ourselves enough?. Asian journal of psychiatry.

[21] Nevitt MC, Cummings SR, Kidd S, Black D (1989). Risk factors for recurrent nonsyncopal falls. A prospective study. Jama.

[22] Foong AW (2007). Rationale and methodology for a population-based study of eye diseases in Malay people: The Singapore Malay eye study (SiMES). Ophthalmic epidemiology.

[23] Lavanya R (2009). Methodology of the Singapore Indian Chinese Cohort (SICC) eye study: quantifying ethnic variations in the epidemiology of eye diseases in Asians. Ophthalmic epidemiology.

[24] Zheng Y (2011). Prevalence and causes of visual impairment and blindness in an urban Indian population: the Singapore Indian Eye Study. Ophthalmology.

[25] Fenwick EK (2017). Vision impairment and major eye diseases reduce vision-specific emotional well-being in a Chinese population. The British journal of ophthalmology.

[26] Ong SY (2012). Visual impairment, age-related eye diseases, and cognitive function: the Singapore Malay Eye study. Archives of ophthalmology (Chicago, Ill.: 1960).

[27] EuroQol–a new facility for the measurement of health-related quality of life. Health policy (Amsterdam, Netherlands) **16**, 199–208 (1990). 10.1016/0168-8510(90)90421-9 10109801

[28] Clemens S, Begum N, Harper C, Whitty JA, Scuffham PA (2014). A comparison of EQ-5D-3L population norms in Queensland, Australia, estimated using utility value sets from Australia, the UK and USA. Quality of life research: an international journal of quality of life aspects of treatment, care and rehabilitation.

[29] Klein BE, Moss SE, Klein R, Lee KE, Cruickshanks KJ (2003). Associations of visual function with physical outcomes and limitations 5 years later in an older population: the Beaver Dam eye study. Ophthalmology.

[30] Luukinen H, Koski K, Kivela SL, Laippala P (1996). Social status, life changes, housing conditions, health, functional abilities and life-style as risk factors for recurrent falls among the home-dwelling elderly. Public health.

[31] Koski K, Luukinen H, Laippala P, Kivela SL (1998). Risk factors for major injurious falls among the home-dwelling elderly by functional abilities. A prospective population-based study. Gerontology.

[32] Pijpers E (2012). Older individuals with diabetes have an increased risk of recurrent falls: analysis of potential mediating factors: the Longitudinal Ageing Study Amsterdam. Age and ageing.

[33] Kamil RJ (2016). Association of Hearing Impairment With Incident Frailty and Falls in Older Adults. Journal of aging and health.

[34] Lopez D (2011). Falls, injuries from falls, health related quality of life and mortality in older adults with vision and hearing impairment–is there a gender difference?. Maturitas.

[35] Hajek A, Konig HH (2017). Falls and subjective well-being. Results of the population-based German Ageing Survey. Archives of gerontology and geriatrics.

[36] Skalska A (2013). The prevalence of falls and their relation to visual and hearing impairments among a nation-wide cohort of older Poles. Experimental gerontology.

[37] Hung CH (2017). Recurrent falls and its risk factors among older men living in the veterans retirement communities: A cross-sectional study. Archives of gerontology and geriatrics.

[38] Zhou J, Qin MZ, Liu Q, Liu JP (2017). Investigation and analysis of osteoporosis, falls, and fragility fractures in elderly people in the Beijing area: a study on the bone health status of elderly people /=80 years old with life self-care. Archives of osteoporosis.

[39] Palagyi, A. *et al*. Visual and refractive associations with falls after first-eye cataract surgery. *Journal of cataract and refractive surgery*, 10.1016/j.jcrs.2017.07.029 (2017). 10.1016/j.jcrs.2017.07.029 29056303

[40] To KG (2014). A longitudinal cohort study of the impact of first- and both-eye cataract surgery on falls and other injuries in Vietnam. Clinical interventions in aging.

