# Supplementary material for: Falls and Recurrent Falls among Adults in A Multi-ethnic Asian Population: The Singapore Epidemiology of Eye Diseases Study
Source: Sci Rep. 2018 May 15;8:7575. doi: 10.1038/s41598-018-25894-8 (PMC5953929; doi:10.1038/s41598-018-25894-8)
Supplement: Supplementary file 1 — Supplementary Information [file 41598_2018_25894_MOESM1_ESM.docx]

**Falls and Recurrent Falls among Adults in A Multi-ethnic Asian Population: The Singapore Epidemiology of Eye Diseases Study**

Wei Dai^1^, Yih-Chung Tham^1^, Miao-Li Chee^1^, Nicholas Y.Q. Tan^1^, Kah-Hie Wong^1,2^, Shivani Majithia ^1^, Charumathi Sabanayagam ^1,3^, Ecosse Lamoureux ^1,3^, Tien-Yin Wong^1,3,4^, Ching-Yu Cheng^1,3,4^

1. Singapore Eye Research Institute, Singapore National Eye Centre, Singapore
2. Department of Ophthalmology, National University Hospital, Singapore
3. Duke-NUS Medical School, Singapore
4. Department of Ophthalmology, Yong Loo Lin School of Medicine, National University of Singapore, Singapore

**Supplementary Table 1.** The associations between demographic, visual impairment, systemic factor, socioeconomic characteristics and frequency of falls.

| **Characteristics** | **Higher frequency of falls** | |
| --- | --- | --- |
|  | **OR * (95% CI)** | **P-value** |
| Age (per decade older) | 1.20 (1.11, 1.30) | <0.001 |
| Female gender | 1.80 (1.55, 2.09) | <0.001 |
| Ethnicity |  |  |
| Malay | Reference |  |
| Indian | 1.13 (0.97, 1.33) | 0.116 |
| Chinese | 1.14 (0.96, 1.35) | 0.138 |
| BMI |  |  |
| Normal | Reference |  |
| Underweight | 0.87 (0.63, 1.20) | 0.389 |
| Overweight | 1.00 (0.87, 1.15) | 0.985 |
| Obese | 1.12 (0.93, 1.34) | 0.240 |
| PVA (based on better eye) |  |  |
| Normal | Reference |  |
| Visually impaired | 1.23 (1.02, 1.48) | 0.028 |
| Systemic disease |  |  |
| Diabetes | 1.21 (1.06, 1.39) | 0.006 |
| Hypertension | 1.08 (0.94, 1.25) | 0.271 |
| Hyperlipidaemia | 0.92 (0.81, 1.05) | 0.225 |
| Chronic kidney disease | 1.01 (0.84, 1.22) | 0.885 |
| Cardiovascular disease | 1.46 (1.21, 1.76) | <0.001 |
| Systemic comorbidities**^#^** |  |  |
| No systemic disease | Reference |  |
| Any 1 systemic disease | 1.14 (0.95, 1.38) | 0.168 |
| Any 2 systemic diseases | 1.10 (0.90, 1.35) | 0.346 |
| ≥ Any 3 systemic diseases | 1.38 (1.12, 1.70) | 0.003 |
| Deafness | 1.21 (0.53, 2.78) | 0.654 |
| EQ-5D score (per SD decrease) | 1.37 (1.30, 1.45) | <0.001 |
| Housing categories |  |  |
| ≥ 5 room public flat/private housing | Reference |  |
| 1-2 room public flat | 1.27 (0.99, 1.65) | 0.065 |
| 3-4 room public flat | 0.94 (0.81, 1.08) | 0.382 |
| Living alone | 1.11 (0.86, 1.45) | 0.428 |
| Education level |  |  |
| No formal education | Reference |  |
| Primary education | 0.95 (0.81, 1.12) | 0.566 |
| Secondary education or above | 1.11 (0.91, 1.34) | 0.296 |
| Monthly income |  |  |
| ≥ 2000SGD | Reference |  |
| < 2000SGD | 1.11 (0.92, 1.34) | 0.284 |
| Current smoker | 0.92 (0.74, 1.13) | 0.418 |
| Alcohol consumption | 1.40 (1.11, 1.78) | 0.004 |

OR: odds ratio, 95% CI: 95% confidence interval, SD: standard deviation, BMI: the body mass index, PVA: presenting visual acuity, EQ-5D: European Quality of Life-5 Dimensions, SGD: the Singapore Dollar.

* Model adjusted for age, gender, ethnicity, presenting visual impairment (based on better eye), BMI, living alone, education, housing categories, income, deafness, EQ-5D score, systemic diseases/comorbidities, current smoker and alcohol consumption.

# The evaluation of systemic comorbidities as exposure of interest was performed in a separate model where diabetes, hypertension, hyperlipidaemia, chronic kidney disease and cardiovascular disease were not included in the model.
